# Effect of N-Ethylmaleimide as a Blocker of Disulfide Crosslinks Formation on the Alkali-Cold Gelation of Whey Proteins

**DOI:** 10.1371/journal.pone.0164496

**Published:** 2016-10-12

**Authors:** Zhao Lei, Xiao Dong Chen, Ruben Mercadé-Prieto

**Affiliations:** Suzhou Key Laboratory of Green Chemical Engineering, School of Chemical and Environmental Engineering, College of Chemistry, Chemical Engineering and Materials Science, Soochow University, Suzhou City, Jiangsu, 215123, P.R. China; Shiraz University, ISLAMIC REPUBLIC OF IRAN

## Abstract

N-ethylmaleimide (NEM) was used to verify that no new disulfide crosslinks were formed during the fascinating rheology of the alkali cold-gelation of whey proteins, which show Sol-Gel-Sol transitions with time at pH > 11.5. These dynamic transitions involve the formation and subsequent destruction of non-covalent interactions between soluble whey aggregates. Therefore, incubation of aggregates with NEM was expected not to affect much the rheology. Experiments show that very little additions of NEM, such as 0.5 mol per mol of protein, delayed and significantly strengthened the metastable gels formed. Interactions between whey protein aggregates were surprisingly enhanced during incubation with NEM as inferred from oscillatory rheometry at different protein concentrations, dynamic swelling, Trp fluorescence and SDS-PAGE measurements.

## Introduction

Whey protein solutions can form stable soluble aggregates [1], for example by heating at low protein concentration [2,3], which can be used to thicken or gel systems at low temperatures after changing one or more parameters, for example after adjusting the pH close to the pI [4]. Here we consider the opposite scenario, whereby we induce the final gelation by increasing the pH to alkaline values. Gelation at acidic pH has been extensively studied compared to alkaline pH. Our interest from alkaline conditions comes from the dissolution of protein hydrogels, used to model dairy fouling, which are industrially cleaned with alkali based solutions [5].

The key mechanistic step(s) that limits the alkaline dissolution at pH ~11.5–13.5 of typical heat-induced whey protein gels is not known. At pH < 11.5 non-covalent interactions are not cleaved in the time frames of hours [6]. At pH >13.5 the ionic concentration is too high, due to the alkali, inhibiting swelling which slows down the detachment of proteins from the gel matrix [7]. At intermediate alkaline pH, it is expected that the breakdown rate of inter-protein non-covalent interactions is important, as studied previously using size exclusion chromatography [6]. However, the study of the formation or destruction of non-covalent crosslinks in highly concentrated protein systems is still very rudimentary, particularly compared to the great advances understanding disulfide crosslinking [8] or heat denaturation [9] of whey proteins.

Swelling experiments of (stranded) whey protein hydrogels at alkaline pH with different [NaCl], which is used to reduce swelling but also stabilizes non-covalent interactions [10], show that there is a dissolution threshold at a certain range of swelling, i.e. at protein concentrations of 7–9 wt% [11]. In order to understand better the stability of non-covalent interactions at alkaline pH, particularly without salts, the alkaline cold gelation of soluble whey protein aggregates was studied in the past [12,13]. It shows a surprising rheology with Sol-Gel-Sol(-Gel) transitions depending on the pH. The first Sol-Gel-Sol transition only involves non-covalent interactions, first they are formed between the soluble aggregates and then they are destroyed at longer times. We have recently confirmed this mechanistic explanation using SDS and NaCl [14]. Another interesting aspect of alkali cold gelation, in addition to the time dependent rheology, is that the elastic modulus at the top of the first gelation transition, caused by non-covalent interactions, is extremely dependent on [WPI] (e.g. power law coefficients of ~25) [13]. Hence, this gelation transition is observed in a very narrow [WPI] range of ~6.8–8 wt%, from a water-like solution into a nice gel, which agrees well with the idea and the [WPI] range observed for the swelling dissolution threshold [11].

Here we continue studying the alkali cold gelation of whey proteins to understand better non-covalent interactions in pH relevant for dissolution and cleaning. In order to further confirm that the initial Sol-Gel-Sol transition does not involve disulfide crosslinking [12], we performed experiments using N-ethylmaleimide (NEM), a compound typically used to block free cysteine groups [15], thus which inhibits the formation of inter-protein covalent crosslinks [4,16,17]. In addition, NEM does not affect the secondary structure or denatures β-lactoglobulin [18,19], hence we expected that NEM would have little or no effect on the alkali cold gelation rheology, but it does to a great extent.

## Materials and Methods

### Sample Preparation

Whey protein isolate (WPI) was purchased from Davisco Foods (Le Seur, MN, USA), with >90% protein. The main chemical of the experiments, N-Ethylmaleimide (NEM, 99% purity), was purchased from J&K Scientific Ltd. Well-homogenized 10 wt% WPI powder solutions in Milli-Q water were heated at 68.5±0.1°C for 2 h or 24 h in a water bath to form soluble aggregates. After cooling down to room temperature, sodium azide (0.05 wt %) was added as a preservative and stored at 4°C. These solutions were used between 2 and 20 days after being prepared. Previous studies suggested that further aggregation still occurred during cold storage [12], hence most experiments were performed within a week. NEM at different molar ratios to that of protein, considering the molecular weight of the major whey protein (β-lactoglobulin, 18.4 kDa), was incubated at 4°C overnight (~12 h) at pH ~7. 8-Anilino-1-naphthalenesulfonate (ANS), NaOH, urea and other chemical reagents used were purchased from Sinopharm (China) of analytical grade and were used as received. The pH of the protein solutions was calculated after experimentally establishing the hydrogen ion equilibrium curve, as described elsewhere [11], with a calculated pH uncertainty of ±0.1.

### Gelation Rheology

**Fig 1:**
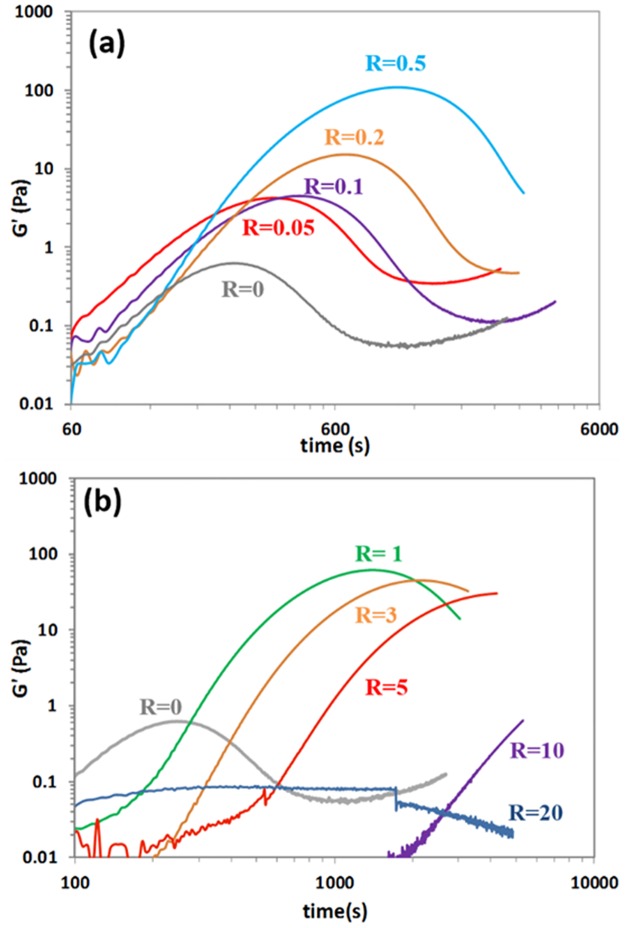
Elastic modulus during the alkali cold gelation of 8 wt% pre-heated WPI solutions (68.5°C for 2 h) at different ratio of [NEM]/[WPI] at pH 11.84 at 25°C, at (a) low NEM concentrations, and at (b) high concentrations.

Alkali cold gelation experiments were performed as described previously [12], briefly: WPI solutions with or without NEM at the desired protein concentration and pH were prepared in a centrifuge tube, mixed by quickly swirling with the tip of the pipette for 10 s, which greatly minimized the formation of bubbles. Then, the solution was pipetted onto the plate of the rheometer. A 40 mm Cone (4°) geometry was used with an oscillatory rheometer, a Kinexus Pro (Malvern, UK) in controlled strain mode, at 0.01 strain and 1 rad/s, in the linear regime. The typical delay time between mixing and data collection was ~40 s, which is included in the figures shown (e.g. Fig 1). Experiments were performed at 25°C.

### Tryptophan Fluorescence

Dynamic and steady state tryptophan fluorescence was measured to study the unfolding of the proteins using a SpectraMax M5 spectrometer (Molecular Devices), excited at 295 nm. In dynamic measurements, the emission at 335, 342, 349 nm was recorded every 30 s. For steady state measurements, the emission spectra were recorded at 330–360 nm. Thirty microliters of 0.5 wt% WPI solutions were incubated in 2.97 mL of 75 mM Na_2_HPO_4_ buffer at different pH.

### Hydrophobicity measurements

Dynamic and steady state hydrophobicity tests were performed using ANS fluorescence. 30 μL of 3 wt % WPI solutions in 2.97 mL of 0.05 mM ANS with 75 mM Na_2_HPO_4_ buffer at different pH, were excited at 375 nm. Fluorescence was recorded in dynamic experiments at 464 and 485 nm every 30 s, similar results were obtained for both wavelengths, only those at 464 nm are shown.

### Zeta Potential

Experiments were performed in a Zetasizer Nano ZS90 (Malvern). All the samples were passed through 0.45 μm PVDF membrane syringe filters to remove the large particles. The zeta potential was calculated from the electrophoretic mobility using the Smoluchowsi model.

### Aggregates Size

The size increase of soluble WPI aggregates with or without NEM at alkaline pH was measured by laser diffraction with a Partica LA-960 (Horiba), in auto iteration mode and using a volume distribution base, and a refractive index RI for the aggregates of 1.5. The swelling of the protein aggregates was measured in 1 wt% WPI solutions with the pH adjusted by adding calculated amounts of 2 M NaOH. Samples were passed through 0.45 μm PDVF syringe filters before analysis. Size measurements were also performed on solubilized aggregates undergoing alkali cold gelation. About 2–3 g of gel were introduced into 50 ml centrifuge tubes with 20 ml of 7 M urea and 50 mM Tris pH 8. Solutions were homogenized with an Ultra-Turrax T18 for 60 s at 18000 rpm, incubated overnight before measurements, and filtered with 5 μm cellulose acetate filters. Size measurements were performed without dilutions in the same urea solutions, using a RI = 1.386 for the solvent.

### SDS-PAGE

The size distribution of protein aggregates during alkali cold gelation (9.45 wt% WPI, pH 11.84) was determined with SDS-PAGE. Twenty microliters of alkali-treated WPI solution or gel were solubilized, after the desired gelation time, in 1 mL of 7 M urea and 50 mM Tris pH 8 buffer, followed by extensive vortex mixing and 15 min in an ultrasonic bath. Samples were incubated overnight at 4°C and analyzed the next day. SDS-PAGE was performed in a XCell SureLock Mini-Cell system (Thermo Fisher Scientific), according to the specification of the manufacturer. NuPAGE 3–8% Tris-Acetate precast gels, Tris-Acetate SDS running buffer and HiMark Pre-stained Protein Standard markers were used for non-reducing conditions. NuPage 4–12% Bis-Tris precast gel, MOPS SDS Running Buffer and SeeBlue Pre-stained Protein Standard marker were used for reducing conditions (50 mM DTT, heated at 70°C for 10 min), everything from Invitrogen.

## Results

### Rheology of alkali cold-gelation with NEM

Fig 1 shows an example of alkali cold-gelation of a WPI solution heated at 68.5°C for 2 h, diluted to 8 wt% WPI and at pH 11.84 without NEM (*R*_NEM/WPI_ = 0). Oscillatory rheology is used to follow the Sol-Gel-Sol transitions that occur within 20 min. The current general explanation of this time-dependent gelation and de-gelation process is, as discussed briefly in the introduction, that the initial soluble disulfide crosslinked aggregates swell and unfold at alkaline pH, allowing new non-covalent interactions to form between different aggregates. However, these new non-covalent interactions are not stable at such high pH and they are slowly degraded with time. According to this simple mechanistic explanation, blocking the free thiol groups in the soluble WPI aggregates should make little difference, particularly on the rheology of cold gelation.

However, Fig 1 shows that increasing the molar ratio of NEM to WPI during incubation, *R*_NEM/WPI_ (using β-lactoglobulin for the molecular weight of the whey mixture), the first gelation step is slower (the elastic modulus *G*’ increases more slowly with time). The time to reach a maximum in *G*’, termed *G*’_max_, and the *G*’_max_ values both increase significantly with *R*_NEM/WPI_ (Table 1), e.g. from *G*’_max_ ~1 Pa without NEM up to ~100 Pa at high *R*_NEM/WPI_ and at a constant [WPI] of 8 wt%. After *G*’_max_, the moduli still decrease at longer times showing that these new interactions formed during gelation are also not stable at high alkaline pH. If NEM is added in large excess, *R*_NEM/WPI_ ≥ 10 (Fig 1(b)), any gelation process is greatly inhibited if not stopped completely, at least in the period studied.

**Table 1:** Characteristic parameters of alkali cold gelation with NEM of pre-heated WPI aggregates with different [NEM]/[WPI] ratios. Errors show one standard deviation.

Pre-heating time (h)	*R*_NEM/WPI_	[WPI]_c.high_ (wt %)	[WPI]_c.low_ (wt %)	[WPI]_c.high_-[WPI]_c.low_ (wt %)	*G*^*'*^_max_ at [WPI]_c.high_ (Pa)	time *G*^*'*^_max_ (s)	time *G*^*''*^_max_ (s)
**2**	0.1	8.7	7.3	1.4	4.2	340 ± 30	300 ± 40
	0.3	8.2	7.0	1.2	2.5	860 ± 90	780 ± 90
	0.5	6.0	5.2	0.8	1.0	1300 ± 100	1270 ± 90
**24**	0.1	6.2	5.2	1.0	0.8	820 ± 40	760 ± 40
	0.3	3.8	3.4	0.4	0.4	1460 ± 40	1340 ± 60
	0.5	3.3	2.9	0.4	0.2	1620 ± 40	1500 ± 60

Similar experiments using soluble WPI aggregates formed for 24 h instead of 2 h, which were more disulfide crosslinked [14], show comparable results with NEM (S1 Fig). The key difference is that for the same [WPI] the *G*’_max_ values for 24 h aggregates are much higher, with the time needed to reach *G*’_max_ being slightly longer as well. Both effects can be explained by the higher incorporation of proteins into aggregates at longer heating times, and by their larger crosslinking degree as shown later on, as was already observed in alkali cold gelation experiments without NEM [14].

### Effect of the protein concentration on the alkali cold-gelation rheology

**Fig 2:**
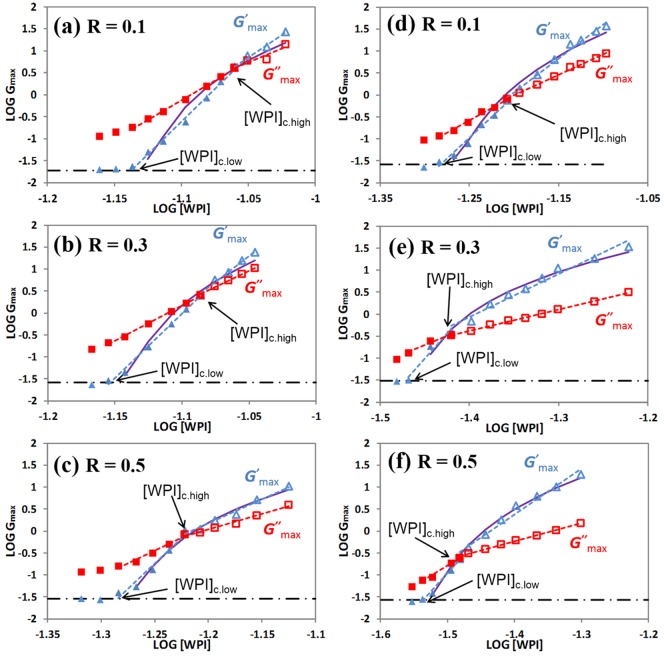
WPI concentrations dependence of *G'*_max_ and *G”*_max_ at different concentration of NEM. (a-c) the aggregates were pre-heated for 2 h at 68.5°C, (d-f) the aggregates were pre-heated for 24 h. Red (*G”*_max_, squares) and blue (*G'*_max_, triangles) straight lines are the best-fit power-law equations considering two regimes. Black dash lines show *G'*_L_ at different conditions; continuous purple lines for *G'*_max_ are the best fit regressions considering the percolation model Eq 3 using [WPI]_c,low_.

An interesting outcome of the Sol-Gel-Sol transitions is that at the peak (*G*’_max_) there is a meta-stable hydrogel stabilized with non-covalent interactions, where their rate of formation equals their rate of breakdown, at least in mechanical terms as observed with rheometry. Non-covalent interactions involve highly cooperative processes; thus they are strongly dependent on the protein concentration [13]. This dependence can be easily studied in alkali cold gelation, as experiments are quick compared to steady state modulus values measured after many hours [20], in particular at concentrations when percolation starts to occur. At high [WPI], the elastic modulus at the peak *G*’_max_ is higher than the viscous modulus (*G*”_max_), a classic benchmark of a gelled system. As the [WPI] is reduced, *G*’_max_ decreases faster than *G*”_max_ yielding a [WPI] where both modulus are equal, termed [WPI]_c,high_ (Table 1). Above and below this concentration, the moduli can be well-described using two power laws, such as

$$G_{max}^{\prime }\;\sim \;{\left[WPI\right]}^{n}$$ (1)

$$G_{max}^{\prime \prime }\;\sim \;{\left[WPI\right]}^{m}$$ (2)

where *n* and *m* are regression parameters obtained experimentally. We conducted experiments at a constant pH of 11.84 and at three different ratios of NEM, from *R*_NEM/WPI_ = 0.1 to 0.5, where a major increase in *G*’_max_ is observed, using aggregates formed for 2 and 24 h. The concentration dependence of the peak moduli at different [WPI], as well as the best-fit Eqs 1 and 2, are shown in Fig 2. Similar results were obtained at *R*_NEM/WPI_ = 0.1 than previously reported without NEM [13], with [WPI]_c,high_ ~8.7 wt% for 2h, as well as for *n* and *m* at the low and high [WPI] range, see Table 2.

**Table 2:** Regression parameters using two power-law regimes, Eqs 1 and 2, and using a percolation model, Eq 3. Uncertainties given are the standard error of the regression parameters.

Pre-heating time (h)	*R*_NEM/WPI_	*n* low [WPI]	*m* low [WPI]	*n* high [WPI]	*m* high [WPI]	t using [WPI]_c.low_	t using [WPI]_p_	[WPI]_p_ (wt %)
**2**	0.1	29.5 ± 1.0	17.7 ± 0.5	20.3 ± 1.7	13 ± 3	3.0 ± 0.3	8.2 ± 0.2	6.0
	0.3	29.2 ± 1.0	16.1 ± 0.5	23.6 ± 1.4	14.8 ± 0.7	3.15 ± 0.19	6.12 ± 0.07	6.4
	0.5	23 ± 3	11.7 ± 1.0	11.0 ± 0.8	7.1 ± 0.4	2.43 ± 0.10	2.56 ± 0.10	5.2
**24**	0.1	19.0 ± 1.3	11.3 ± 0.6	15.5 ± 0.7	9.4 ± 0.2	3.11 ± 0.14	5.06 ± 0.06	4.7
	0.3	23 ± 5	8.3 ± 1.5	9.8 ± 0.6	4.86 ± 0.10	2.20 ± 0.13	3.85 ± 0.11	2.9
	0.5	24 ± 2	9.4 ± 0.8	15.1 ± 1.0	4.18 ± 0.12	2.49 ± 0.08	3.01 ± 0.04	2.8

At higher NEM ratios some differences become apparent, the cross point concentration occurs at lower [WPI]_c,high_ values, ~3 wt% lower at *R*_NEM/WPI_ = 0.5, and the value of the cross point *G*’_max_ is also reduced, to one fourth by *R*_NEM/WPI_ = 0.5. There are few differences on the power law coefficients *n* and *m*, considering that in some experimental sets the available [WPI] range is small and not many data points are used. Note that the *G*_max_ values are obtained at much longer times at higher *R*_NEM/WPI_. Considering all this, the only salient difference is the lower *m* values for 24 h aggregates at *R*_NEM/WPI_ 0.3 and 0.5. For example, the ratio *n*/*m* is fairly constant at ~1.6 when using only alkali, regardless of the type of aggregates used [13], but it increases up to ~3 at *R*_NEM/WPI_ = 0.5.

For comparison we have also fitted the *G'*_max_ data to a percolation model [13,21] using

$$G_{max}^{\prime }-G_{L}^{\prime }\;\sim \;{\left(\left[WPI\right]/{\left[WPI\right]}_{p}\;-1\right)}^{t}$$ (3)

where *t* is the critical exponent related to the geometry of the underlying lattice and [WPI]_p_ is the percolation concentration. If we fix [WPI]_p_ = [WPI]_c,low_, the values of *t* calculated are similar for both types of aggregates at 2.2–3.1, as observed in alkali cold gelation without NEM [13,14]. If we allow [WPI]_p_ to be estimated by an optimization method, the calculated *t* values vary significantly up to physically unrealistic values (Table 2), as seen previously [13]. Eq 3 only describes well the *G'*_max_ data at *R*_NEM/WPI_ = 0.5, otherwise two power laws are preferred.

The effect of adding NEM on the [WPI] dependence of the peak moduli is comparable to the effect of increasing the pre-heating time, from 2 to 24 h hours, briefly shown here, but discussed in more detail elsewhere [14]. For instance, at the same [WPI] both *G'*_max_ and *G”*_max_ are much higher in 24 h aggregates, and the time to reach both peak values is much longer. [WPI]_c,high_, and *G'*_max_ at that concentration are much lower in 24 h aggregates. There is little difference in the coefficients *n* and *m*, considering experimental variability, but they are generally lower in the 24 h aggregates. The similarity between NEM and increasing the aggregation time is surprising considering that the major contribution of the later is to increase the disulfide crosslinking of the soluble aggregates. Hence, the rheological profiles shown in Fig 1, as well as the [WPI] effect in Fig 2, both suggest that NEM is enhancing the crosslinking of the soluble protein aggregates to a large extent (at least as much as the pre-heating time), which is clearly not what was initially expected.

### Effect of NEM on the first Sol-Gel transition

As discussed in the introduction, alkali cold-gelation is an interesting system to study non-covalent interactions between aggregates in alkaline pH relevant to dissolution. Hence, the fact that small additions of NEM affect the rheology so extensively, compared to any other chemical added to date (NaOH, NaCl, SDS), was considered worthy of further study; especially if it conflicts with the current mechanistic explanation of alkali cold gelation. Further experiments were performed at *R*_NEM/WPI_ = 0.5, the lowest NEM ratio where major rheological changes are observed.

**Fig 3:**
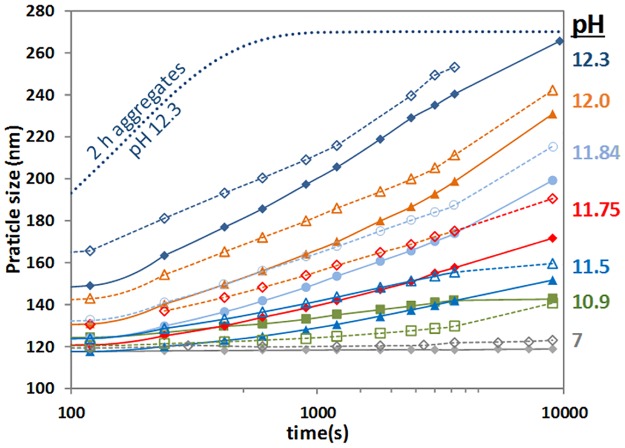
The diameter profiles of 1 wt% WPI 24 h aggregates at different pH (unbuffered) and room temperature, without NEM (empty symbols, dashed lines) and with NEM at *R*_NEM/WPI_ = 0.5 (solid symbols, continuous lines). Dotted line shows comparable results from the literature for 2 h preheated WPI aggregates at pH 12.3 [12].

The first gelation step involves the unfolding of the aggregates or its constituent proteins as the pH in suddenly increased. The reason why the soluble aggregates can still denature extensively is the relatively low temperature used in the pre-heating process, 68.5°C [22,23]. As the aggregates unfold, their size also increases significantly, both processes facilitating the formation of new non-covalent crosslinks. Hence, it could be speculated that NEM affected this swelling process following the significant delays observed in the first Sol-Gel transition (S1 Fig), compared to experiments without NEM [13]. The dynamic swelling of 24 h aggregates was followed at different pH, with and without NEM, results shown in Fig 3. At pH < 11.5, there is little size increase in agreement with previous studies that show that at such alkaline pH non-covalent interactions in whey protein aggregates are stable [6], unlike in un-aggregated β-lactoglobulin where the secondary and tertiary structure is lost at lower alkaline pH [24]. In such conditions, classic Sol-Gel transitions are observed. At pH > 11.5, where Sol-Gel-Sol transitions occur, the dynamic swelling as well as the final particle size both increase with pH. In swelling experiments with NEM the measured sizes are systematically lower, typically by ~15 nm at the same time. This difference is not large, but it is statistically significantly considering that the variability of duplicate or triplicate experiments was ±5 nm. If we compare the present swelling results using 24 h aggregates with those reported previously for 2 h aggregates [12], the 24 h aggregates clearly swell much more slowly; e.g. at >1000 s there was little size change with 2 h aggregates (dotted line Fig 3), but with the 24 h ones a steady state size is still not observed at high pH after 3 h.

Clearly, the slower swelling of the 24 h aggregates compared to the 2 h ones corresponds well with their longer time needed to reach *G'*_max_ (Table 1 and [14]). In more crosslinked aggregates, it is intuitive that they require longer times to unfold or swell. Similar conclusion but to a lower extent can be reached at *R*_NEM/WPI_ = 0.5, suggesting again that NEM treated aggregates are more crosslinked or stable.

**Fig 4:**
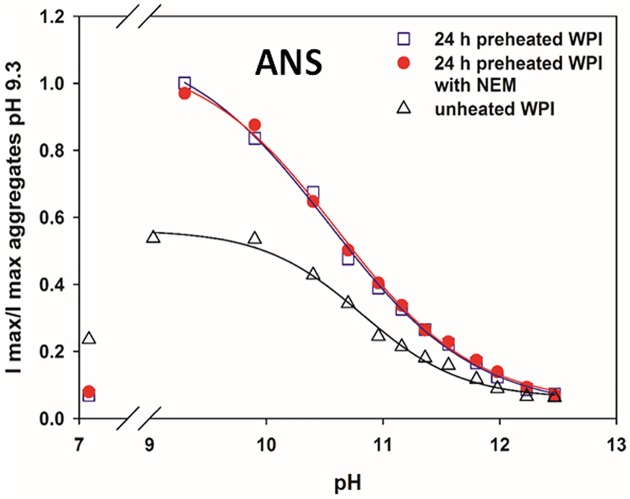
Relative maximum fluorescence intensity after 1 h incubation at different pH, using unheated WPI, and 24 h pre-heated WPI with (*R*_NEM/WPI_ = 0.5) and without NEM. Data normalized with the fluorescence intensity of aggregates without NEM at pH 9.3. Final [WPI] = 0.03 wt%, [ANS] = 0.05 mM, excitation wavelength λ_ex_ = 295 nm, emission wavelength λ_em_ = 464 nm. Lines show the best-fit sigmoidal curves to determine the pK_a_.

We then used the hydrophobic probe ANS to follow up for the first time the alkali denaturation of the soluble aggregates. At neutral pH, ANS binds more extensively in the native protein compared to the 24 h aggregates, provably due to the limited access of buried sites [25]. As the pH is increased to ~9 the ANS fluorescence intensity increases significantly due to the more open conformation of βLg after the Tanford transition at pH >7.5, improving the binding of ANS inside the βLg β-barrel calix [26] (Fig 4). The fluorescence of the aggregates at pH ~9 is about twice that of unheated whey, suggesting that in the aggregates there are more accessible hydrophobic sites for ANS to bind [27]. As the aggregates or the native proteins unfold with pH and the buried binding sites are exposed, the ANS fluorescence intensity decreases, in the case of the aggregates to the same level than at neutral pH.

The alkali induced denaturation of the main whey protein βLg can be well described considering the acid base equilibria of the ionizable amino acids, in particular that of lysines due to its relative large amount in βLg, with an estimated pK_a_ of 10.6 [24]. The ANS transition at alkaline pH in unheated WPI solutions also shows such sigmoidal transition with a calculated pK_a_ ~10.5. This transition does not change significantly for the 24 h aggregates, with or without NEM, with pK_a_ ~10.7. This is surprising because previously it was found that such base transitions, using different techniques, are shifted about 1 pH unit higher in aggregates than in unheated whey [6], as shown subsequently for Trp fluorescence.

The steady state ANS values do not show any effect due to the incubation by NEM. However, some differences are apparent when checking the dynamics of the ANS fluorescence decrease at alkaline pH, S2 Fig. The fluorescence intensity in WPI-NEM aggregates decreases more slowly during the initial ~250 s, approaching then the steady state value, than without NEM. Hence, in the initial stages of the Sol-Gel transition, WPI-NEM aggregates are slightly more difficult to unfold, which is consistent with the swelling data of Fig 3.

**Fig 5:**
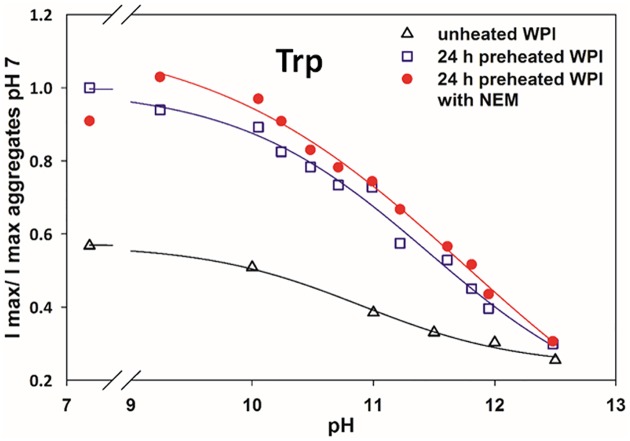
Relative maximum tryptophan fluorescence intensity of native WPI after 1 h at different pH, using unheated WPI, and 24 h pre-heated WPI with (*R*_NEM/WPI_ = 0.5) and without NEM. Data normalized with the fluorescence intensity of aggregates without NEM at neutral pH. Final [WPI] = 0.005 wt%, excitation wavelength λ_ex_ = 295 nm; the maximum emission intensity λ_em_ between 330–360 nm is used.

The denaturation of whey protein aggregates was also monitored using tryptophan (Trp) fluorescence (Fig 5). As reported previously [27], the Trp fluorescence intensity in whey protein aggregates is much higher than in native whey. Similar than discussed for ANS, as the β-barrel calix of βLg unfolds at higher alkaline pH, the steady state fluorescence intensity, measured after 1 h, decreases as shown previously for heat denaturation [28]. This transition with pH is known to occur in aggregates at pK_a_ values higher than in the native proteins, here at 11.45±0.2 and ~10.9 respectively, in agreement with previous studies [6]. Measurements using WPI-NEM show that at the same pH the steady state Trp fluorescence is higher, yet they reach the same value at pH ~12.5 when aggregates are extensively denatured, as expected. Hence, the calculated pK_a_ for WPI-NEM is higher at 11.75. This further suggests that the NEM treated aggregates are more stable, becoming more difficult to unfold, maybe due to more extensive crosslinking. The dynamics of the Trp fluorescence decrease are shown in S3 Fig, but unlike for ANS there are no salient differences in the rates, only in the final steady state values.

**Fig 6:**
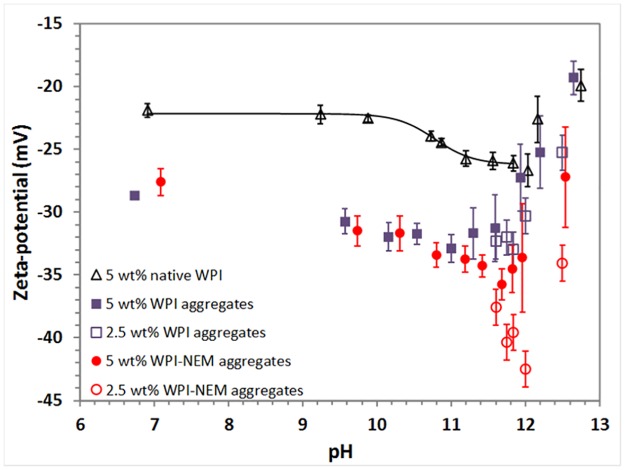
Zeta-potential of native WPI and 2 h WPI aggregates with (*R*_NEM/WPI_ = 0.5) and without NEM at different pH conditions. Error bars show the SD of 4–7 measurements within 15 min.

The zeta potential of whey protein aggregates is more negative than in unheated protein solutions (Fig 6), in agreement with previous studies [29]. In native whey we can also observe for the first time the base denaturation transition as a decrease of the zeta potential with an estimated pK_a_ value of ~10.8. At pH >12 fluctuations with time were observed, making the results less reliable. In addition, the viscosity increases noticeably due to aggregation which causes an increase of the zeta potential at pH > 12 [30]. Using 2 h WPI aggregates, there is a mild decrease of the zeta potential as the pH is increased, but it increases at pH >12 due to the same reasons than for native whey (note that the protein concentration used is much higher than for the particle size measurements in Fig 3). Measurements at a [WPI] of 5 wt% did not show any difference due to NEM, yet measurements at 2.5 wt%, when new protein interactions become more difficult, show statistically lower values at pH > 11.5 with NEM. The diluted NEM data suggest a transition that may related to that observed with Trp fluorescence, that with a pK_a_ ~11.5, but no such thing is observed without NEM (maybe explained because of the faster gelation without NEM than with NEM). On the other hand, the slightly lower zeta-potential values with NEM occur at the same pH where alkali-cold gelation occurs, which could be related to the slower modulus increase with NEM due to extra negative repulsion. An additional explanation of the lower zeta-potentials with NEM can be the alkylation of lysines with NEM, as discussed later on, which would reduce the number of positive charges.

**Fig 7:**
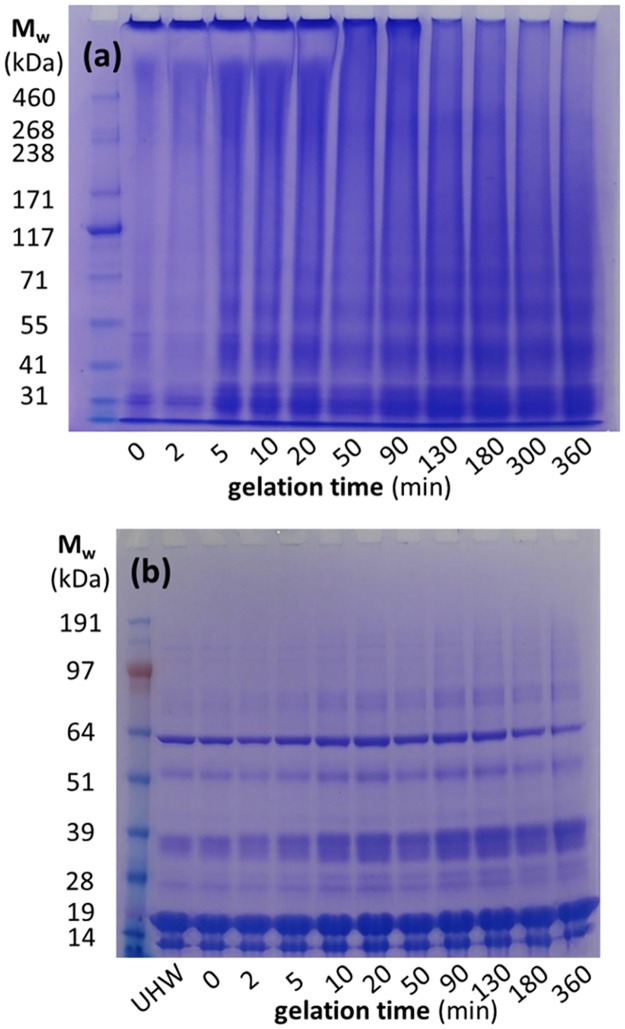
(a) Non-reducing and (b) reducing SDS-PAGE of solubilized solutions or gels during the alkali cold gelation of 9.45 wt% 2h WPI aggregates with NEM (*R*_NEM/WPI_ = 0.5) at pH 11.84 and at different gelation times, and for unheated WPI (UHW).

The experiments described so far try to understand what happens to diluted whey protein aggregates that mostly do not interact with other aggregates, except in the case of the zeta potential, due to the low [WPI] used. In order to investigate what happens at much higher [WPI], when the Sol-Gel-Sol transitions occur, we solubilized and quenched the gels in a buffer with 7 M urea. The SDS-PAGE gels during alkali cold gelation of 2 h and 24 h aggregates is shown in S4 and S5 Figs, respectively, and agree well with previous results [13]. The main difference of 24 h aggregates is the much higher initial degree of disulfide crosslinking compared to the 2 h ones. In fact, almost all the 2 h aggregates can diffuse inside the PAGE. However, if the same 2 h aggregates are incubated with NEM at *R*_NEM/WPI_ = 0.5, most of the protein aggregates cannot diffuse inside the PAGE (Fig 7). During alkali cold gelation at pH 11.84, these very large aggregates are slowly broken down, with a clear decrease between 20 and 50 min, in the same time frame where the rheological de-gelation step occurs (Fig 1(a)).

**Fig 8:**
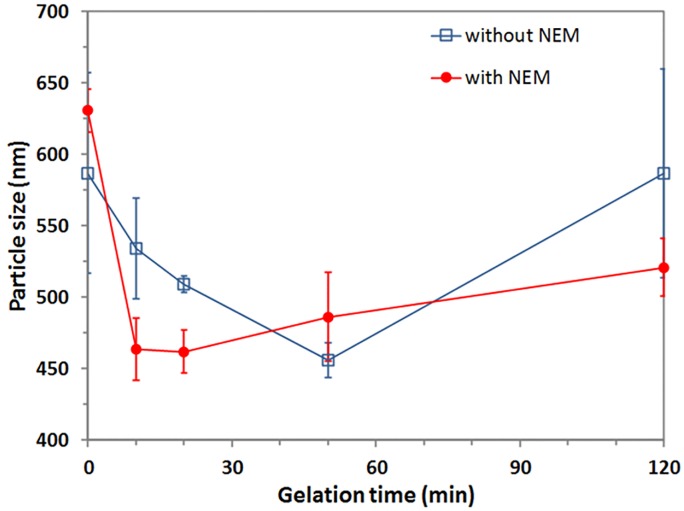
Particle size of aggregates solubilized and measured in 7 M urea and 50 mM Tris buffer pH 8, during the alkali cold gelation of 9.45 wt% 2 h WPI aggregates at pH 11.84 at different gelation times. The measurement [WPI] was ~1 wt%. Samples were filtered to remove >2 μm impurities. Error bars show one SD.

The very different size of aggregates with and without NEM according to SDS-PAGE was not apparent using particle size measurements shown in Fig 8. This could be due the inability of the particle sizer to measure small size differences in challenging measuring conditions (e.g. 7 M urea), or because there is indeed not much size difference because of NEM, but internal rearrangements within aggregates. We note that we also failed to measure a size difference between the 2 h and 24 h aggregates using laser diffraction, despite the clear difference in SDS-PAGE [14].

## Discussion

NEM has been extensively used in studies trying to understand the chemical interactions during the gelation of whey and many other protein systems, with the sole intended purpose to inhibit disulfide crosslinking [4,16]. However, NEM can do other things than intended, even at neutral pHs, as exemplified by the work of Havea *et* al. [31] in the head induced gelation of whey protein gels. Searching back in the literature, it was long known that NEM can react with several amino acids other than just blocking free cysteines. For instance, Cys and His promote the polymerization of NEM at alkaline pH, resulting in a pink color, as observed in this study (S6 Fig) [32]. However, the most interesting reaction is the alkylation of the amino group of Lys and the imidazole group of His [33]. These reactions occur very slowly during incubation at neural pH, but quickly at alkaline pH when the amino groups in Lys are deprotonated and can react with NEM. It is plausible from Fig 1(b) that if NEM is in excess during alkaline cold gelation, extensive alkylation of the aggregates can inhibit the formation of new non-covalent interactions, and therefore gelation is delayed. However, the major rheological effects observed at low NEM ratios, such as 0.5, are unlikely caused by alkylation reactions once the pH becomes alkaline, but by modifications of the soluble aggregates during incubation with NEM, before the pH change.

One possibility that should be considered is the formation of covalent crosslinks through non-natural amino acids, as in lysinoalanine [34], due to the presence of NEM. Such new crosslinks would not be expected to be reduced with DTT, and therefore should be visible as dimmers in SDS-PAGE using reducing conditions. Fig 7(b) shows a small but quantifiable increase in the dimmer bands of βLg-βLg (~37 kDa), from ~15% (which come presumably from significant casein impurities) to 20%, and βLg-BSA (~82 kDa), from ~5% to 10%, after >10 min. However, such an increase is also observed in the results without NEM, hence it should be due to the alkali cold-gelation process or other reasons [35], such as by the DTT itself [36], but not due to NEM.

The strong effect of NEM at very small concentrations brings to mind the comparable effect of divalent salts, such as calcium, in gelation and swelling. Calcium is known to bind locally to carboxylates, electrostatically shielding charges much more efficiently, and therefore at much lower concentrations than NaCl for example [37]. We checked if small additions of calcium, at molar ratios between 0.1 and 1, could induce similar rheological changes as NEM in the alkali cold-gelation, results shown in S7 Fig. Increasing the amount of calcium does not change *G'*_max_, or the time needed to reach *G'*_max_. The only salient effect is the inhibition of the de-gelation step at high calcium concentrations. Non-covalent interactions become more stable at alkaline pH in the presence of calcium [7], which is the same effect observed when adding NaCl during alkali cold gelation but at much higher salt concentrations [14].

The SDS-PAGE of the NEM treated WPI suggests an increase in the disulfide crosslinking in the aggregates, which is considered highly unlikely following the extensive literature proving the efficiency of NEM blocking free thiol groups [15,19,38,39]. One hypothesis is that NEM stabilizes the soluble aggregates during the typical run of a SDS-PAGE, but different strategies were tried to destroy the WPI-NEM aggregates without success (S8 Fig). The only (non-reducing) chemical found that could break these large WPI-NEM clusters that cannot go into the Tris-Acetate PAGE is NaOH itself. After >2 h of alkali cold gelation at pH 11.84, the bands of the 2 h WPI-NEM gels (Fig 7(b)) resemble the bands of the 24 h aggregates without NEM (S5(b) Fig), but are clearly more crosslinked than the corresponding 2 h aggregates without NEM (S4(b) Fig). This suggests, again, more covalent crosslinking because of NEM, which is hard to explain. Yet, as the disulfide crosslinked aggregates without NEM are fairly stable with time at pH 11.84, the relatively fast destruction of the WPI-NEM aggregates at the same pH may suggest a non-covalent nature for the new crosslinks.

Understanding the chemical interactions in protein gels has been an important area of research in food science and engineering. In this study we used NEM to further validate that disulfide crosslinking is not important in the first Sol-Gel-Sol transition observed in the alkali cold gelation of whey proteins. Yet incubation of soluble aggregates with small amounts of NEM delay and strengthen the alkali gelation transition, meanwhile a de-gelation step is still observed, highlighting the still weak nature of the new chemical interactions formed between aggregates. The rheological experiments shown in Fig 1, as well as those at different [WPI] in Fig 2, suggest a larger initial degree of crosslinking (or molecular weight) of the soluble aggregates with NEM, exactly as observed due to the effect of the pre-heating time. This predicted higher extent of crosslinking is clearly observed with SDS-PAGE, but not with particle size measurements. Swelling and Trp fluorescence measurements show that aggregates incubated with NEM are slightly more stable or crosslinked, supporting the main conclusion that the key modifications occur during incubation, not during alkaline cold gelation. No conclusive additional information is obtained to explain the rheological effects of NEM using ANS or the zeta potential. Hypothetical explanations of the effect of NEM must consider that it has the opposite effect than intended, somehow promoting additional crosslinking during overnight incubation at low temperatures, the nature of which is not even clear. If NEM is in large excess, the likely extensive alkylation of lysines at alkaline pH delay and eventually inhibit the formation of a gel, even if the initial aggregates are more crosslinked. Our study highlights the difficulties to study experimentally the chemical interactions, particularly of a non-covalent nature, between protein aggregates to form percolating hydrogels. In addition, it confirms the growing evidence in the literature that NEM, the most extensively used chemical to block the formation of disulfide crosslinks, has many unintended side-effects in aggregates and gels, which clearly deserves to be investigated further.

## Supporting Information

S1 Fig Comparison of the alkali cold gelation with NEM using WPI aggregates formed after heating for 2 h and 24 h at 68.5°C. In order to provide comparable experiments, different [WPI] are shown, 7.5 wt% for 2 h, and 5 wt% for 24 h aggregates. Note that for *R*_NEM/WPI_ = 0 and 0.1 for 24 h, the [WPI] is lower than [WPI]_c.low_, so no gelation transition is observed.
(TIF)

S2 Fig Dynamic hydrophobility measurements using ANS fluorescence. Values normalized with the maximum intensity at pH 9.3 without NEM. Empty symbols are used without NEM, solid symbols with NEM at *R*_NEM/WPI_ = 0.5. 24 h WPI aggregates were used, at a final [WPI] = 0.03 wt%, [ANS] = 0.05 mM, λ_ex_ = 295 nm, λ_em_ = 464 nm.
(TIF)

S3 Fig Dynamic tryptophan fluorescence measurements. Values normalized with the maximum intensity at pH 7 without NEM. Empty symbols are used without NEM, solid symbols with NEM at *R*_NEM/WPI_ = 0.5. 24 h WPI aggregates were used, at a final [WPI] = 0.005 wt%, fixed the λ_ex_ = 295 nm, recorded the maximum intensity of λ_em_ between 330–360 nm.
(TIF)

S4 Fig SDS-PAGE of solubilized solutions or gels during the alkali cold gelation of 2 h WPI aggregates without NEM incubation. **(a)** Non-reducing and **(b)** reducing. Gelation conditions: Gelation conditions: 9.45 wt% WPI at pH 11.84 using 2 h pre-heated aggregates at 68.5°C.
(TIF)

S5 Fig SDS-PAGE of solubilized solutions or gels during the alkali cold gelation of 24 h WPI aggregates without using NEM incubation. **(a)** Non-reducing and **(b)** reducing SDS-PAGE of solubilized solutions or gels during the alkali cold gelation of 24 h WPI aggregates without using NEM incubation. Gelation conditions: 9.45 wt% WPI at pH 11.84 using 24 h pre-heated aggregates at 68.5°C.
(TIF)

S6 Fig Color change in due to NEM at alkaline pH. (a) Before, and (b) after pH change. 8 wt% WPI aggregates (preheated at 68.5°C for 2 h) with different ratio of [NEM] and [WPI] *R*_NEM/WPI_ at a constant pH 11.84.
(TIF)

S7 Fig Alkali cold gelation profiles with calcium chloride. 8 wt% 2 h preheated WPI aggregates with different molar ratios of calcium at pH 11.84 (continuous lines). Control experiments without changing the pH, only by adding CaCl_2_, are shown as dashed lines of the same color. Diamond symbols at the bottom show an unmodified whey protein aggregate solution for comparison.
(TIF)

S8 Fig Non-reducing SDS-PAGE of 2 h WPI aggregates incubated with NEM at *R*_NEM/WPI_ = 0.5 (left line). Instead of the normal SDS solution used to prepare the SDS gels, the aggregates were incubated in a urea 7 M, or 0.5 wt% SDS, or in 5 M GuHCl. These solvent where also used to solubilize the proteins during alkali cold gelation at pH 11.78, results are shown at two different gelation times. GuHCl caused the protein aggregates to precipitate, and nothing diffused into the PAGE.
(TIF)
