# Supplementary figures and images for: Effect of N-Ethylmaleimide as a Blocker of Disulfide Crosslinks Formation on the Alkali-Cold Gelation of Whey Proteins
Source: PLoS One. 2016 Oct 12;11(10):e0164496. doi: 10.1371/journal.pone.0164496 (PMC5061392; doi:10.1371/journal.pone.0164496)

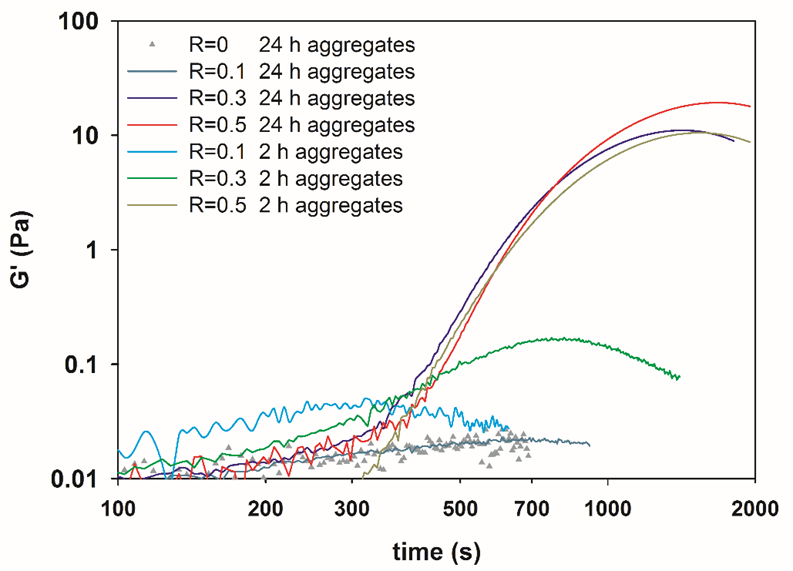

Supplement: S1 Fig — In order to provide comparable experiments, different [WPI] are shown, 7.5 wt% for 2 h, and 5 wt% for 24 h aggregates. Note that for RNEM/WPI = 0 and 0.1 for 24 h, the [WPI] is lower than [WPI]c.low, so no gelation transition is observed. (TIF) [file pone.0164496.s001.tif]

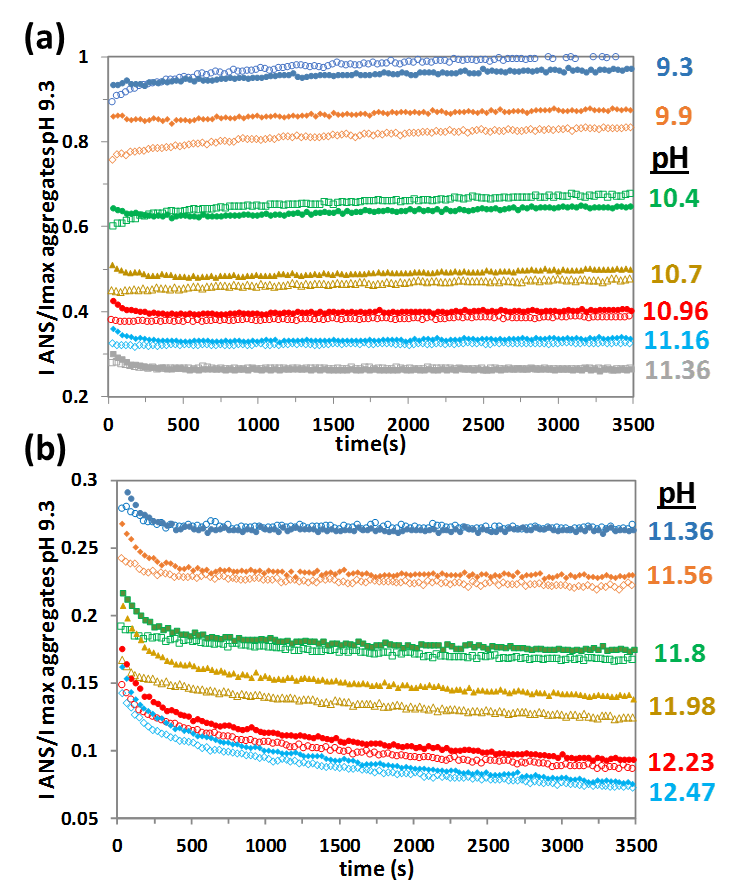

Supplement: S2 Fig — Values normalized with the maximum intensity at pH 9.3 without NEM. Empty symbols are used without NEM, solid symbols with NEM at RNEM/WPI = 0.5. 24 h WPI aggregates were used, at a final [WPI] = 0.03 wt%, [ANS] = 0.05 mM, λex = 295 nm, λem = 464 nm. (TIF) [file pone.0164496.s002.tif]

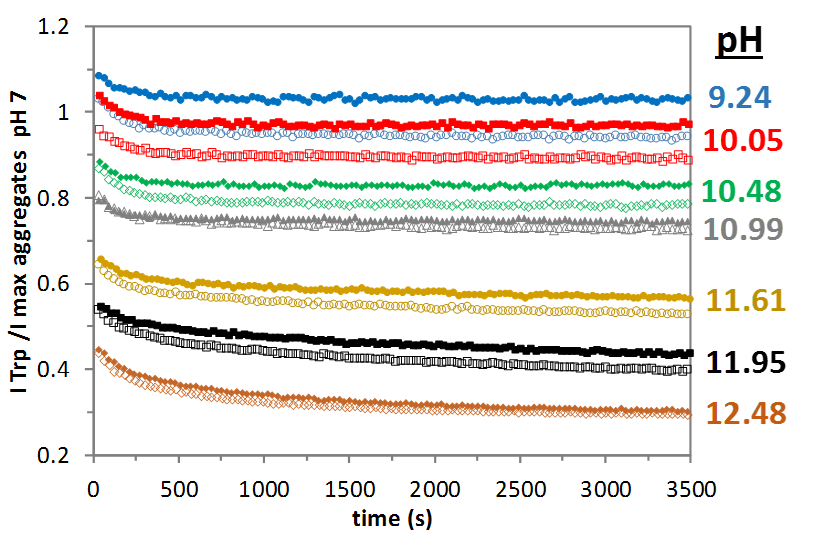

Supplement: S3 Fig — Values normalized with the maximum intensity at pH 7 without NEM. Empty symbols are used without NEM, solid symbols with NEM at RNEM/WPI = 0.5. 24 h WPI aggregates were used, at a final [WPI] = 0.005 wt%, fixed the λex = 295 nm, recorded the maximum intensity of λem between 330–360 nm. (TIF) [file pone.0164496.s003.tif]

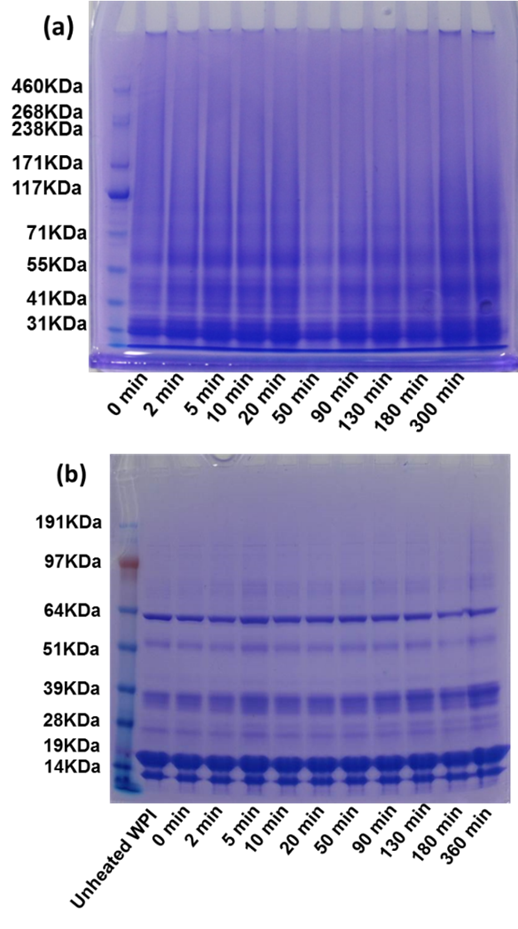

Supplement: S4 Fig — (a) Non-reducing and (b) reducing. Gelation conditions: Gelation conditions: 9.45 wt% WPI at pH 11.84 using 2 h pre-heated aggregates at 68.5°C. (TIF) [file pone.0164496.s004.tif]

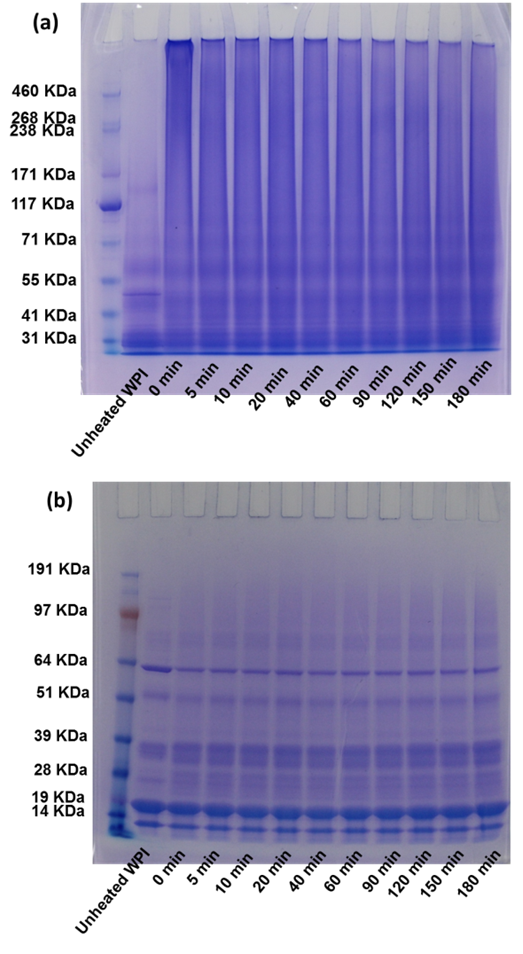

Supplement: S5 Fig — (a) Non-reducing and (b) reducing SDS-PAGE of solubilized solutions or gels during the alkali cold gelation of 24 h WPI aggregates without using NEM incubation. Gelation conditions: 9.45 wt% WPI at pH 11.84 using 24 h pre-heated aggregates at 68.5°C. (TIF) [file pone.0164496.s005.tif]

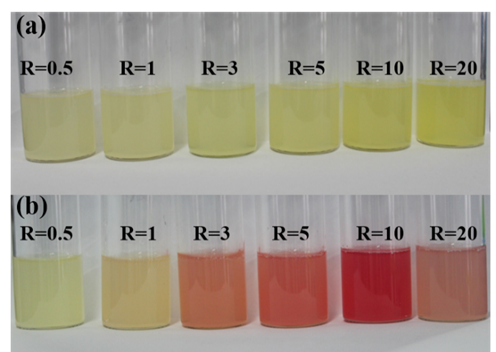

Supplement: S6 Fig — (a) Before, and (b) after pH change. 8 wt% WPI aggregates (preheated at 68.5°C for 2 h) with different ratio of [NEM] and [WPI] RNEM/WPI at a constant pH 11.84. (TIF) [file pone.0164496.s006.tif]

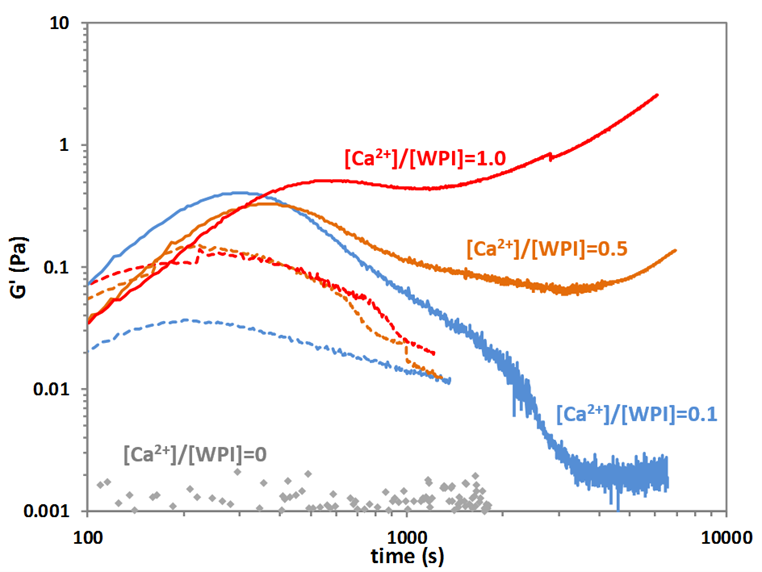

Supplement: S7 Fig — 8 wt% 2 h preheated WPI aggregates with different molar ratios of calcium at pH 11.84 (continuous lines). Control experiments without changing the pH, only by adding CaCl2, are shown as dashed lines of the same color. Diamond symbols at the bottom show an unmodified whey protein aggregate solution for comparison. (TIF) [file pone.0164496.s007.tif]

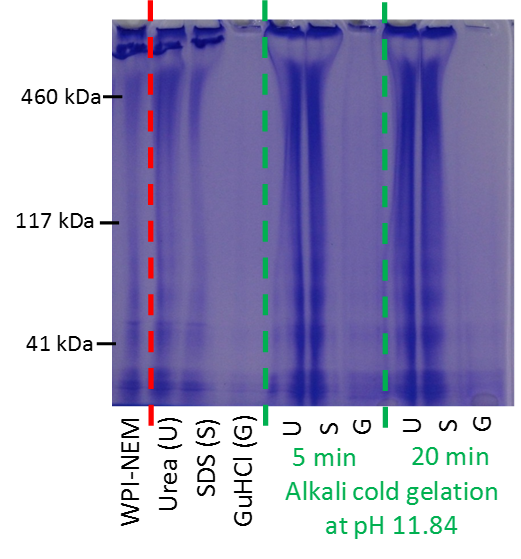

Supplement: S8 Fig — Instead of the normal SDS solution used to prepare the SDS gels, the aggregates were incubated in a urea 7 M, or 0.5 wt% SDS, or in 5 M GuHCl. These solvent where also used to solubilize the proteins during alkali cold gelation at pH 11.78, results are shown at two different gelation times. GuHCl caused the protein aggregates to precipitate, and nothing diffused into the PAGE. (TIF) [file pone.0164496.s008.tif]
